# Supplementary material for: Structural basis of peptidoglycan synthesis by E. coli RodA-PBP2 complex
Source: Nat Commun. 2023 Aug 24;14:5151. doi: 10.1038/s41467-023-40483-8 (PMC10449877; doi:10.1038/s41467-023-40483-8)

Structural basis of peptidoglycan synthesis by *E. coli* RodA-PBP2 complex

Rie Nygaard[1], Chris L.B. Graham[2], Meagan Belcher Dufrisne[3], Jonathan D. Colburn[2,4], Joseph Pepe[1], Molly A. Hydorn[5], Silvia Corradi[1,6], Chelsea M. Brown[2,4], Khuram U. Ashraf[1], Owen N. Vickery[2,4], Nicholas S. Briggs[2], John J. Deering[2], Brian Kloss[7], Bruno Botta[6], Oliver B. Clarke[1,8], Linda Columbus[3,*], Jonathan Dworkin[5,*], Phillip J. Stansfeld[2,4,*], David I. Roper[2,*] and Filippo Mancia[1,*]

[1]Department of Physiology and Cellular Biophysics, Columbia University Irving Medical Center, New York, NY 10032, USA.

[2]School of Life Sciences, University of Warwick, Coventry, CV4 7AL, UK

[3]Department of Chemistry and Department of Molecular Physiology and Biological Physics, University of Virginia, Charlottesville, VA, 22904, USA

[4]Department of Chemistry, University of Warwick, Coventry, CV4 7AL, UK

[5]Department of Microbiology and Immunology, Columbia University Irving Medical Center, New York, NY, 10032, USA

[6]Faculty of Pharmacy and Medicine, Sapienza University of Rome, Rome, Italy

[7]New York Consortium on Membrane Protein Structure, New York Structural Biology Center, 89 Convent Avenue, New York, NY, 10027, USA

[8]Department of Anesthesiology, Columbia University Irving Medical Center, New York, NY, 10032, USA

Supplementary Figure 1

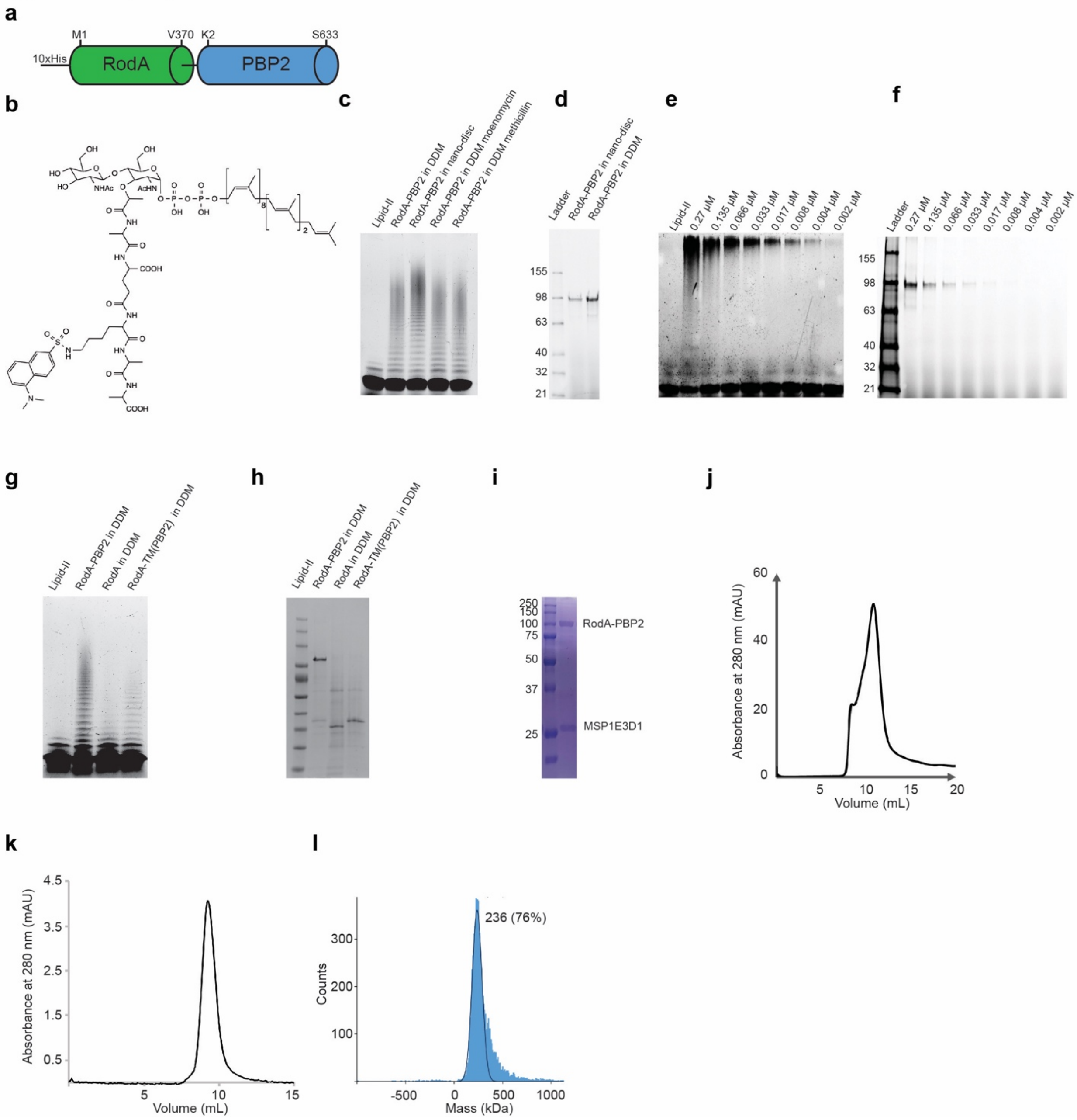

Supplementary Figure 1 | RodA-PBP2 purification and functional characterization.

a) Construct design for the RodA-PBP2 fusion with a His tag genetically fused to the N-terminus of RodA. **b**) Chemical structure of dansyl lysine Lipid II. The fluorescent dansyl-label is attached at position 3 of the peptide stem. **c**) Electrophoresis analysis of Lipid II polymerization products of RodA-PBP2 in detergent and nanodisc. In the last two lanes shown, we added either

moenomycin or methicillin at a concentration of 5.4 μM. **d**) Visualization of RodA-PBP2 protein used for polymerization assays. Bocillin staining confirms integrity of PBP2 transpeptidase active site and reflects protein folding. **e**) Electrophoresis analysis of Lipid II polymerization products of RodA-PBP2 with varying amounts of RodA-PBP added. **f**) Visualization of RodA-PBP2 protein used for polymerization assays using bocillin staining of SDS-PAGE gel. **g**) Electrophoresis analysis of Lipid II polymerization for full length RodA-PBP2 fusion, RodA alone (terminating at residue 373 of RodA) and RodA-TM(PBP2) (terminating at residue 47 of PBP2) in detergent. **h**) SDS-PAGE gel of purified RodA-PBP2 and truncated constructs**.** **i**) SDS-PAGE gel of the RodA-PBP2 complex after final SEC. **j**) SEC elution profile of RodA-PBP2 in nanodisc at the final purification step. **k**) SEC elution profile of fraction 11 from the final SEC purification step, used for freezing on grids, re-run on analytical SEC. **l**) Mass photometry profile of the sample used for freezing on grids showing that 76% of the particles fit under the peak at 236 KDa corresponding to one RodA-PBP2 fusion complex in nanodisc.

Supplementary Figure 2

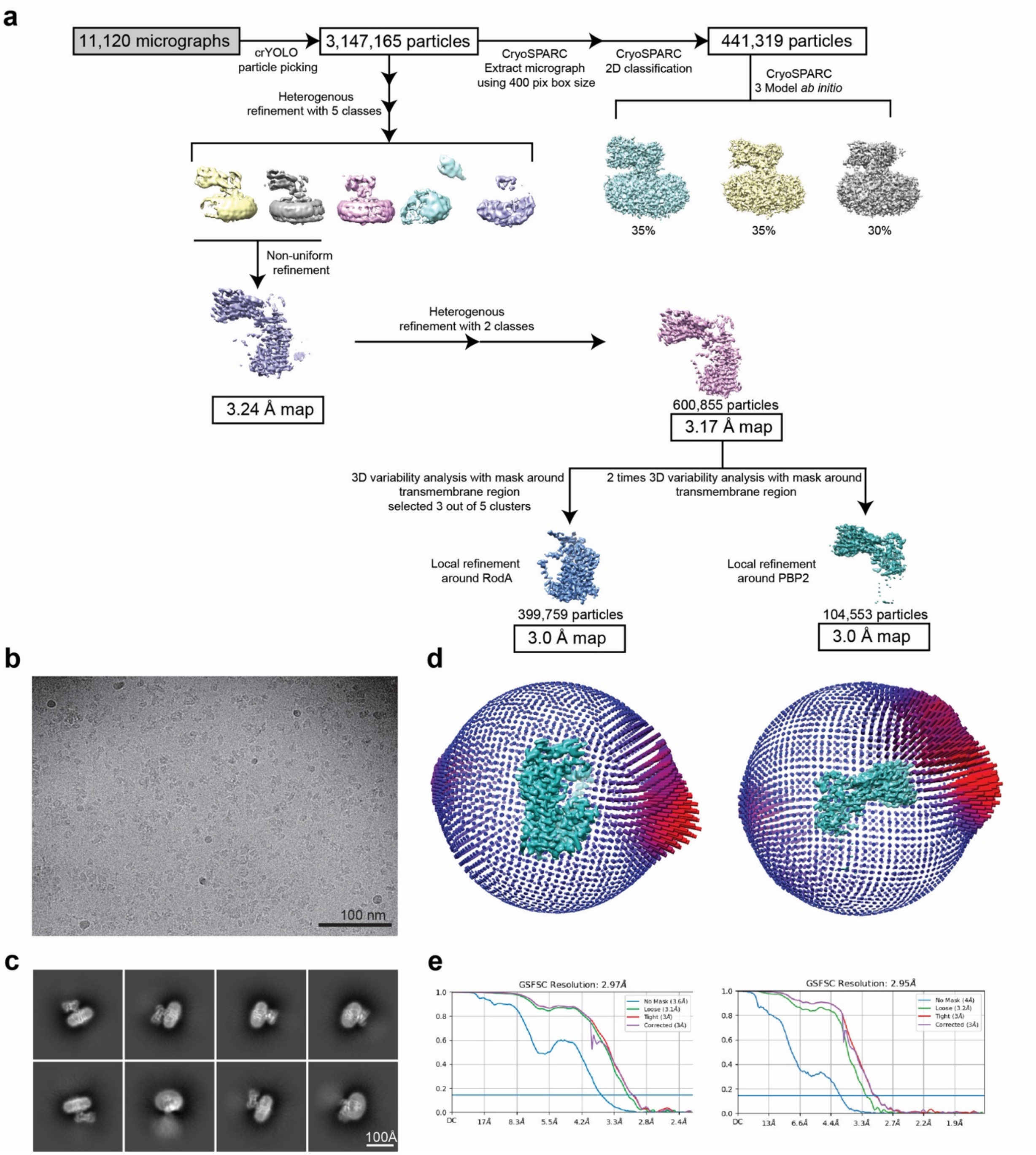

Supplementary Figure 2 | cryo-EM analysis of the RodA-PBP2 complex in nanodisc. a) Flow chart of cryo-EM image acquisition and data processing of the RodA-PBP2 complex. **b**) Representative micrograph. 11,120 micrographs were collected in total. **c**) Representative 2D class averages from cryoSPARC 2D classification. **d**) Euler angle distribution of all particles used in

the final map reconstruction. Final map shown in green. Each orientation is represented by a cylinder, with each cylinder's height and color (from blue to red) proportional to the number of particles for that specific direction. **e**) Fourier shell correlation (FSC) curves for the local refinement of the transmembrane region to the right and the periplasmic region to the left.

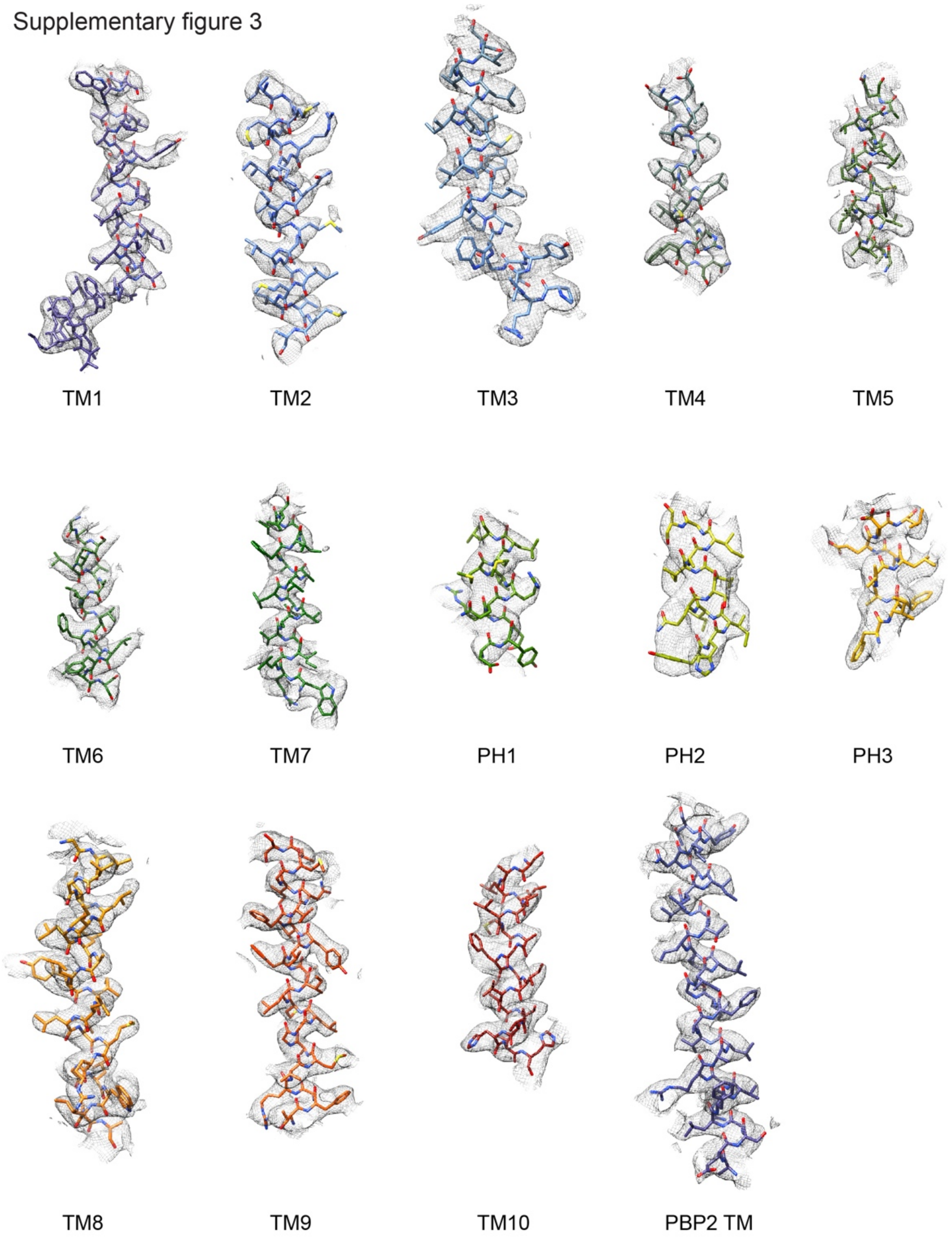

Supplementary Figure 3 | Fit of cryo-EM density with model.

Cryo-EM densities (mesh) are superimposed on secondary structure elements from RodA. The model is rendered in sticks using the color scheme as in Fig. 1d and e.

Supplementary Figure 4

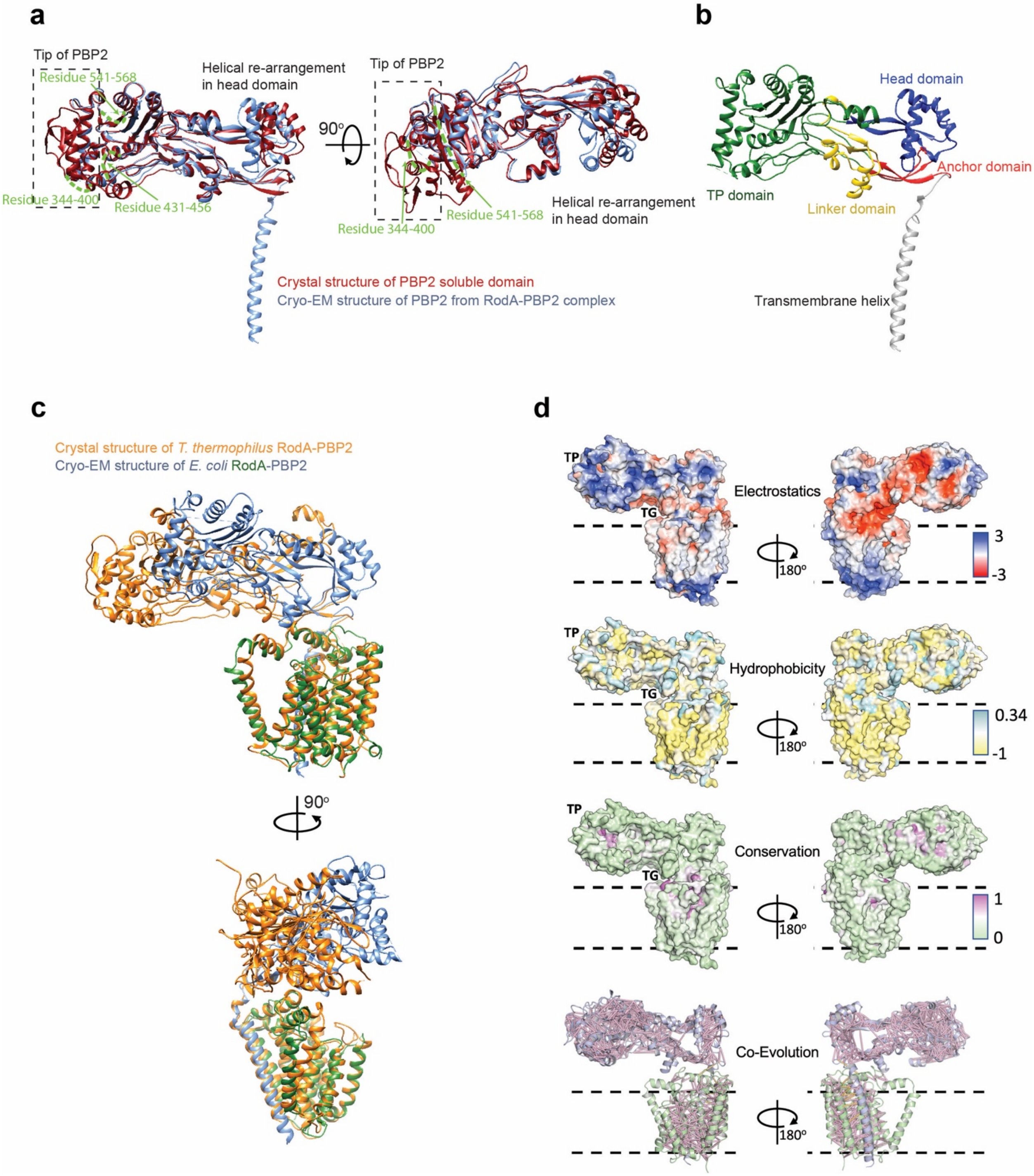

Supplementary Figure 4 | Structural features of the RodA-PBP2 complex. a) Comparison of the previously published X-ray crystallography structure of PBP2 without the transmembrane helix (PDB ID 6G9S) in red and PBP2 from our current cryo-EM structure of the RodA-PBP2 complex in blue. The tip of PBP2 looks to be partially unfolded in our structure and could not be resolved.

b) PBP2 from the RodA-PBP2 complex colored by sub-domain as in Levy et al[1]. **c**) Comparison of our structure of RodA-PBP2 with the one from *T. thermophilus*. The *T. thermophilus* structure is shown in orange and the *E. coli* one in blue (PBP2) and green (RodA). **d**) RodA-PBP2 rendered in surface representation coloured by electrostatic potential on a range of ±5 kBT/e, by Wimley-White hydrophobicity, on a cyan (very hydrophilic) to gold (very hydrophobic) scale, by conservation on a green (no conservation) to purple (absolute conservation) scale and by co-evolutionary analysis calculated using MapPred[2] and mapped onto the cryo-EM structure of RodA-PBP2, using a threshold of 0.272. Intramolecular predicted contacts between Cα are shown as purple dashes and intermolecular predicted contacts are shown as yellow dashes.

Supplementary Figure 5

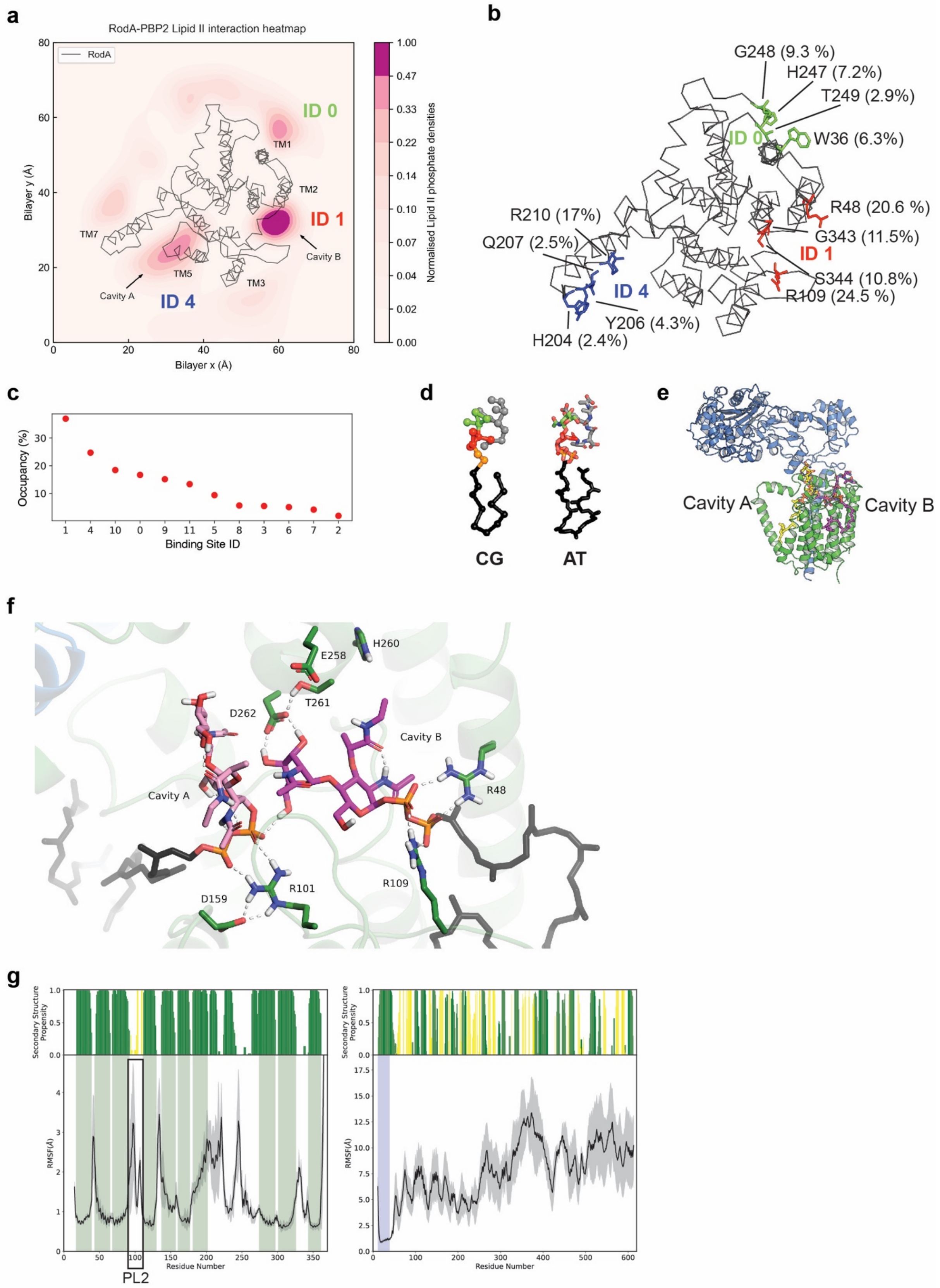

Supplementary Figure 5 | Docking and MD simulation analysis. a) Density plots from CG MD simulations of Lipid II binding, with binding site IDs noted from PyLipID analysis. **b)** PyLipID occupancy analysis of the three main binding sites to RodA, with the top 4 residues with the greatest occupancy shown for each site. **c)** Ranking of the binding site IDs based on occupancy. Binding site ID 10 is not shown in **a** or **b**, as it is predominantly bound to the PBP2 head, not RodA. **d)** CG and atomistic (AT) representations of Lipid II. **e)** Representations of the bound Lipid II molecules to both cavity A and B. **f**) Close-up of the RodA active site from a minimized DFTB cluster model. Lipid II molecules bound in cavites A and B as well as key residues are shown as sticks and labelled. Not shown are the backbone atoms of the peptide fragments constituting the cluster. Hydrogen-bonds are indicated with white dashes. **g**) Root Mean Square Fluctuation (RMSF) analysis of the Cα trace of RodA-PBP2. The RMSF measurements were averaged across 3 repeats of 1 μs simulation. The grey shading refers to the standard deviation across the repeats. The bar graph on top of the RMSF plot shows the α-helix propensity over the course of the simulation as with the green bars. Black box highlights the residues in PL2.

Supplementary figure 6

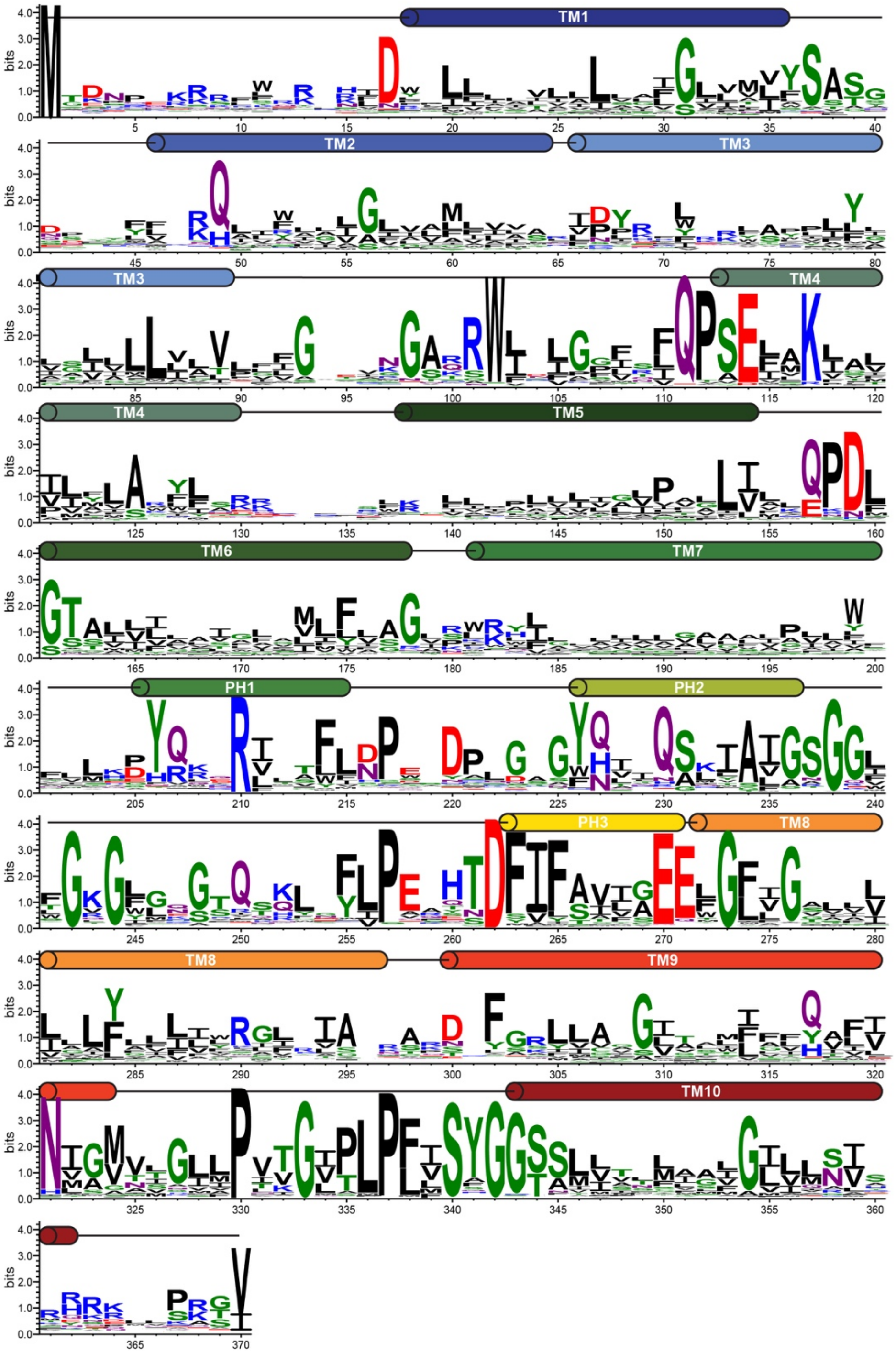

Supplementary Figure 6 | Conserved residues of RodA. Weblogo analysis of RodA created by Weblogov3[3] using the *E. coli* RodA sequence with MMseqs2[4] to search for homologous sequences within both UniRef100 and the environmental sequence databases. This resulted in ~14,000 sequences. Positions of RodA α-helical regions shown above weblogo coloured as in Fig. 1d. Key to residues colors: Black, hydrophobic; purple, hydrophilic; red, acidic; blue, basic; green hydroxyl & glycine.

Supplementary figure 7

a

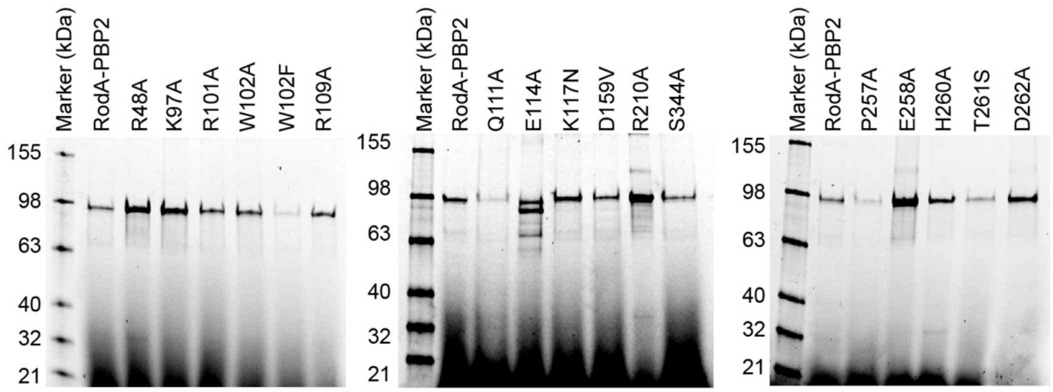

b

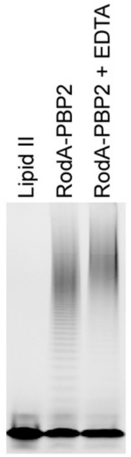

Supplementary Figure 7 | Expression of mutant protein and metal-ion independence. a) Visualization of RodA-PBP2 protein mutants used for polymerization assays in Fig. 2e and 4c. Bocillin staining confirms integrity of PBP2 transpeptidase active site and approximates protein folding. **b**) Electrophoresis analysis of Lipid II polymerization products of RodA-PBP2 with and without EDTA (35 mM).

Supplementary Figure 8

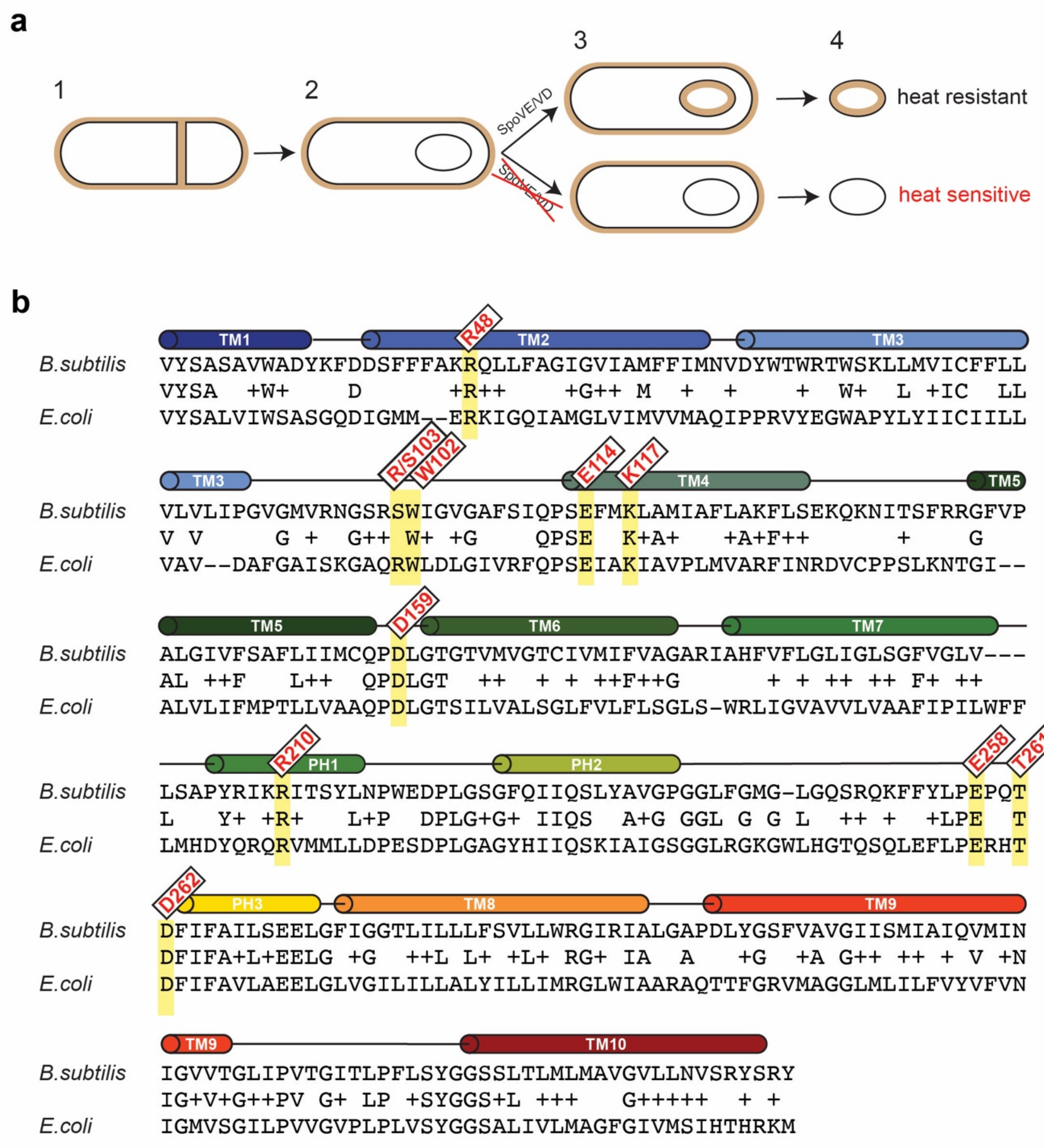

Supplementary Figure 8 | Genetic analysis of SEDS-PBP function. a) Assay of sporulation-specific SpoVE-SpoVD function. (1) asymmetric division during sporulation; (2) formation of a membrane bound forespore compartment; (3) synthesis of spore PG is dependent on SpoVE/VD; (4) heat sensitivity of spores lacking SpoVE/VD. **b)** BLAST alignment of *E. coli* RodA fusion and *B. subtilis* SpoVE fusion.

Supplementary Figure 9

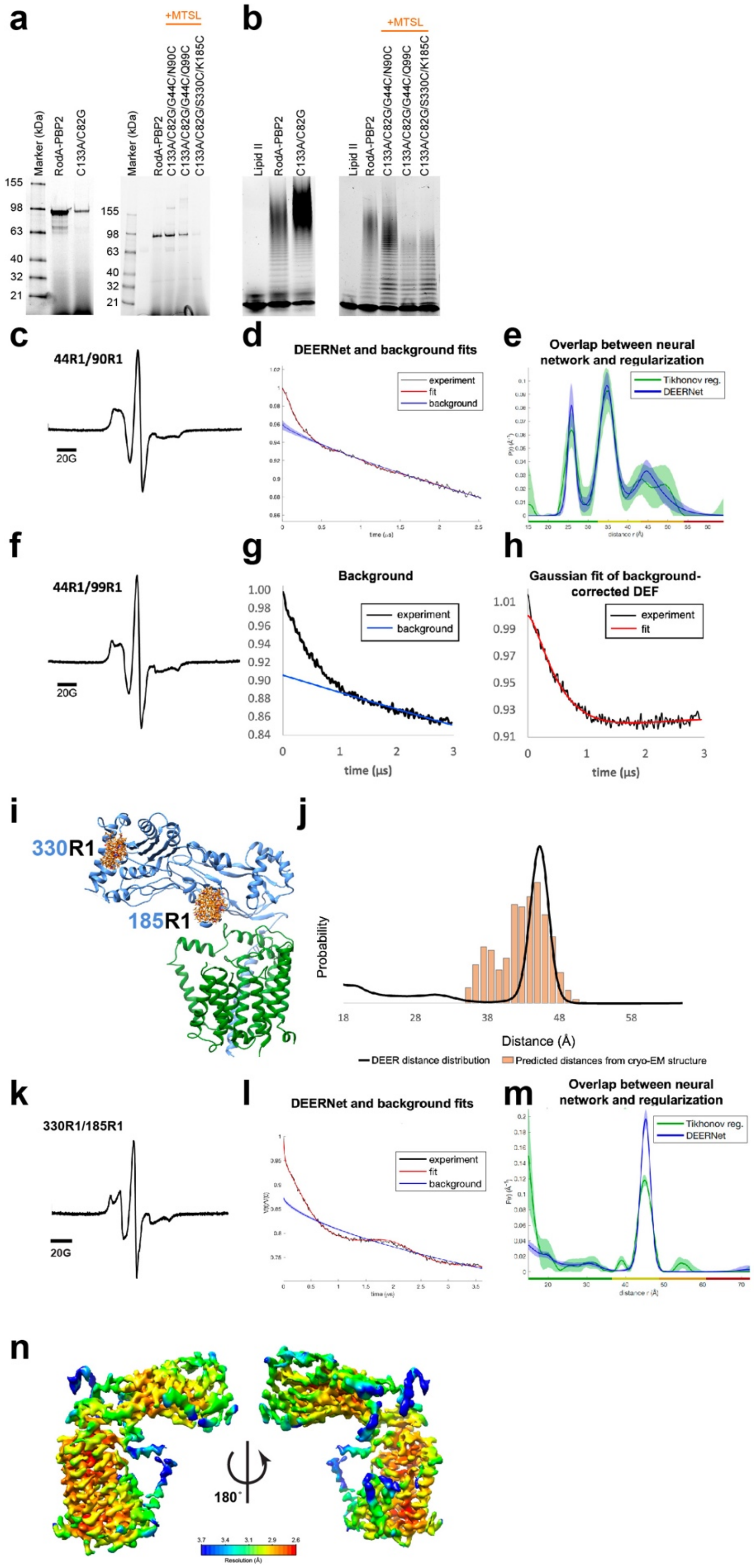

Supplementary Figure 9 | Dynamics of RodA-PBP2. a) Visualization of RodA-PBP2 protein mutants used for used for the DEER experiments presented in Fig. 3 and 5 by Bocillin staining, confirming integrity of PBP2 transpeptidase active site and approximates protein folding. For the S300A mutant, there is no evidence of bocillin binding because S300 is its binding site in PBP2[1]. **b**) Electrophoresis analysis of Lipid II polymerization products of RodA-PBP2 for the cysteine-free background and double-cysteine constructs used for DEER experiments presented in Fig. 3 and 5 with spin label (MTSL). **c**) Continuous wave (CW) EPR spectra of spin labeled Gly44R1/Asp90R1 RodA-PBP2 mutant (44R1/90R1). Background estimation and fits of the resulting experimental DEER dipolar evolution function (DEF) (**d**) and resulting DEER distance distributions (comparison of Tikhonov regularization and DEERNet neural network) for 44R1/90R1 RodA-PBP2 calculated with the DEERNet extension of DEERAnalysis[5] (**e**). The DEERNet distance distribution was used for main figures and analysis. **f**) CW EPR spectra of spin labeled Gly44Cys/Gln99Cys RodA-PBP2 mutant (44R1/99R1). **g**) Background estimation of the resulting DEER data and **h**) Gaussian fit of the background-corrected DEF for 44R1/99R1 RodA-PBP2 done with DEERAnalysis[6]. **i**) Sterically allowed rotamers of nitroxide spin labels (R1) are illustrated for Lys185R1/Ser330R1 on the cryo-EM structure (orange sidechains). **j**) Resulting DEER distance distributions (Lys185R1/Ser330R1; solid black line) are compared to predicted distance distributions (orange bars) based on the sterically allowed rotamers shown on the structure. **k**) Continuous wave (CW) EPR spectra of spin labeled Lys185R1/Ser330R1 RodA-PBP2 mutant (185R1/330R1). **l**) Background estimation and fits of the resulting experimental DEER dipolar evolution function (DEF) and **m**) resulting DEER distance distributions (comparison of Tikhonov regularization and DEERNet neural network) for Lys185R1/Ser330R1

RodA-PBP2 calculated with the DEERNet extension of DEERAnalysis[5]. **n**) Local resolution display of orthogonal view of RodA-PBP2 from non-uniform refinement without a mask.

Supplementary figure 10

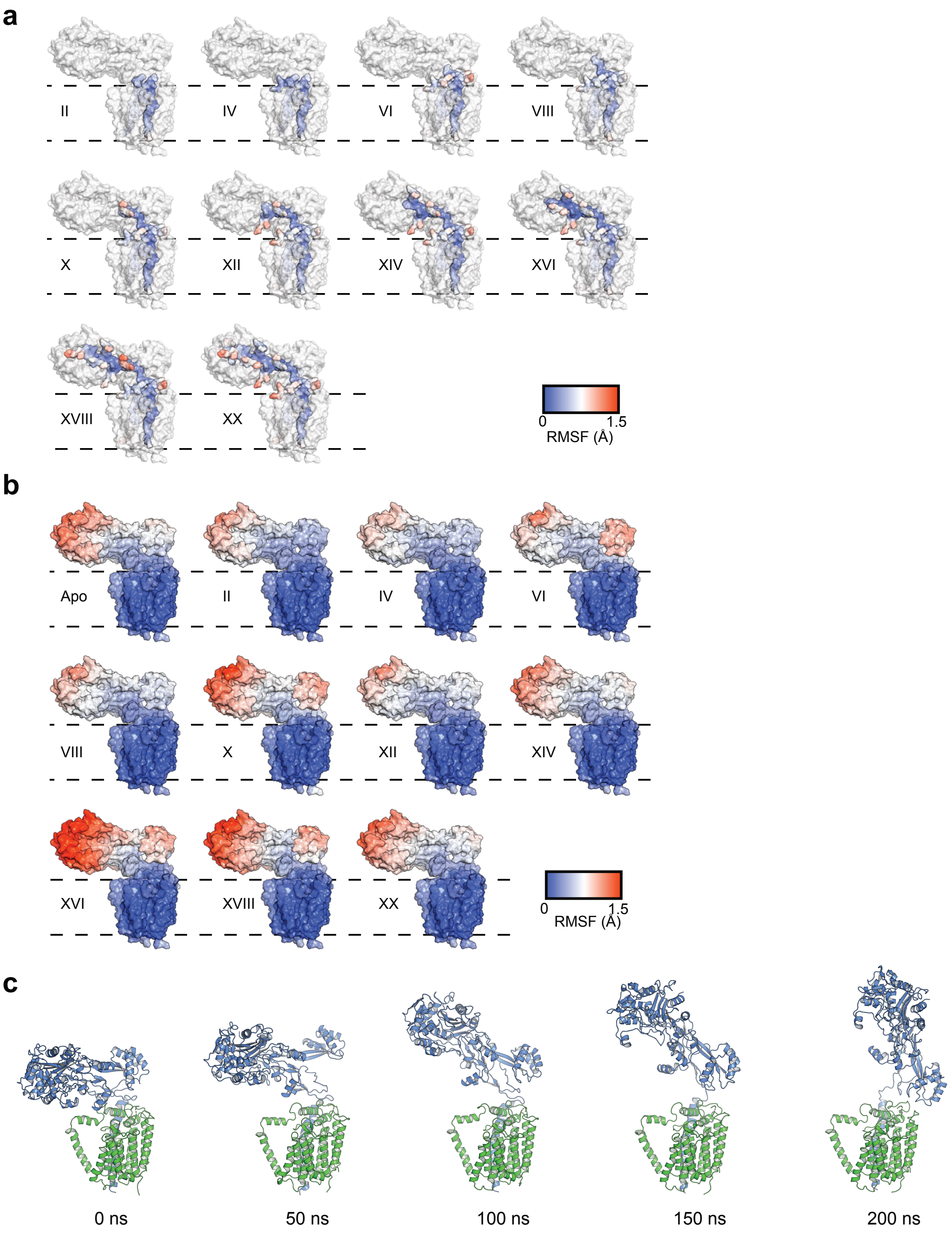

Supplementary Figure 10 | Analysis of MD simulations with varying length of the glycan strand. a) RodA-PBP2 is shown as a light grey surface representation with Lipid II-Lipid XX shown as surface representation colored by RMSF from blue to red. The polysaccharides are stably coordinated within the binding groove whereas the pentapeptide stem is more mobile. **b**) RodA-PBP2 shown as a surface representation colored by RMSF from blue to red. **c)** Snapshots from a single MD simulation of RodA-PBP2 with Lipid II and Lipid XVIII bound. The protein is shown in cartoon representation at 0, 50,100,150 and 200 ns timepoints.

Supplementary Table 1. SEDS-PBP fusion proteins

Bacterial species	SEDS UniProt ID	PBP UniProt ID	MW (kDa)
Enterococcus faecalis	Q820T8	Q836V7	126.8
Enterococcus faecalis	Q820T8	Q830D1	122.8
Klebsiella pneumoniae	A0A0H3GLD1	A0A0W8ASI8	112.0
Escherichia coli	**P0ABG7**	**P0AD65**	**111.8**
Streptococcus pneumoniae	A0A062WN05	P14677	128.2
Streptococcus pneumoniae	A0A062WN05	A0A0Y0HFB8	119.7
Streptococcus pneumoniae	A0A062WN05	A0A0H2URT5	126.5
Escherichia fergusonii	B7LLH8	B7LLH7	111.7

Supplementary Table 2. Summary of Cryo-EM Data Collection and Model Refinement

	**RodA-PBP2** (EMD-XXX) (PDB XXX)
**Data collection and processing**	
Magnification	105,000
Voltage (kV)	300
Electron exposure (e-/Å^2)	58.5
Exposure time (s)	2.5
Dose rate (e-/pixel/s)	16.1
Nominal defocus range (μm)	-2.5 to -1
Defocus range (μm)	-2.4 to -1.5
Pixel size (Å)	0.83
Symmetry imposed	C1
Number of micrographs	11,120
Initial particle images (no.)	3,462,335
Final particle images (no.)	399,759 (Transmembrane region local refinement) and 104,553 (PBP2 periplasmic domain local refinement)
Map resolution (Å)	2.97 (Transmembrane region local refinement) and 2.95 (PBP2 periplasmic domain local refinement)
FSC threshold	0.143
**Refinement**	
Map sharpening *B* factor (Å^2)	-82.4 (Transmembrane region local refinement) and -84.3 (PBP2 periplasmic domain local refinement)
Residue range	RodA (9 to 93 and 109 to 365) and PBP2 (10-343, 401-430, 457-540 and 569-612)
Model composition	
Non-hydrogen atoms	6,475
Protein residues	833
Ligands	0
R.m.s. deviations	
Bond lengths (Å)	0.004
Bond angles (°)	0.725
Validation	
MolProbity score	2.03
Clashscore	9.52
Poor rotamers (%)	1.16
Ramachandran plot	
Favored (%)	92.33
Allowed (%)	7.55
Disallowed (%)	0.12

Supplementary Table 3. Table summarizing genetic and biochemical activity assays for SpoVE-SpoVD and RodA-PBP2 fusions

Mutant	*In vivo* Sporulation	*In vitro* RodA GT activity
R48A	strong (~5%) (R48A)	NDA (Fig. 2)
K97A	ND	WTA (Fig. 2)
R101A	no defect (S103A)	LA (Fig. 2)
W102A	severe (W104A)	LA (Fig. 2)
W102F	severe (W104F)	WTA (Fig. 2)
R109A	ND	WTA (Fig. 2)
E114A	severe (E116A)	WTA (Fig. 2)
K117N	severe (K119N)	WTA (Fig. 2)
D159V	severe (D163V)	LA (Fig. 2)
R210A	severe (R212A)	LA (Fig. 2)
P257A	severe (P258A)	NDA (Fig. 4)
E258A	moderate (~10%)(E259A)	WTA (Fig. 4)
H260A	ND	WTA (Fig. 4)
T261S	moderate (~10%)	LA (Fig. 4)
D262A	severe (D263A)	NDA (Fig. 4)
S344A	ND	WTA (Fig. 2)

In vivo Sporulation: Sporulation efficiency was assayed by heat resistance relative to wild type. "severe" is when sporulation efficiency of is less than 10^{-7}; “strong” is <10%; “moderate” is 10%-50%; no defect is >50%. Mutant is in reference to the *E. coli* RodA sequence; corresponding residues in *B. subtilis* SpoVE are noted in brackets.
ND: not determined.

In vitro RodA GT activity: WTA is the GT activity corresponding to wild type (without mutations) RodA-PBP2 fusion (green), NDA is no detectable GT activity (red), LA is Low GT activity judged as <50% activity of wild type RodA-PBP2 fusion (amber).

Supplementary Table 4.

Mutant	Primer Sequences
**G44C**	Forward: AGC GGT CAG GAT ATT TGC ATG ATG GAG CGT AAA<br>Reverse: TTT ACG CTC CAT CAT GCA AAT ATC CTG ACC GCT
**R48A**	Synthesized by Azenta Biosciences - Genewiz. Nucleotide sequence changed from CGT to GCT.
**C82G**	Forward: GCT GGG CCC CCT ATC TCT ATA TCA TCG GTA TTA TTT TGC TGG TGG CGG TAG ATG<br>Reverse: CAT CTA CCG CCA CCA GCA AAA TAA TAC CGA TGA TAT AGA GAT AGG GGG CCC AGC
**D90C**	Forward: TTG CTG GTG GCG GTA TGT GCT TTC GGT GCC ATC<br>Reverse: GAT GGC ACC GAA AGC ACA TAC CGC CAC CAG CAA
**K97A**	Forward: CGG TAG ATG CTT TCG GTG CCA TCT CTG CAG GTG CTC AAC GCT GGC TGG ACC TCG<br>Reverse: CGA GGT CCA GCC AGC GTT GAG CAC CTG CAG AGA TGG CAC CGA AAG CAT CTA CCG
**R101F**	Forward: CAA TAC CGA GGT CCA GCC AGA ATT GAG CAC CTT TAG AGA TGG CAC<br>Reverse: GTG CCA TCT CTA AAG GTG CTC AAT TCT GGC TGG ACC TCG GTA TTG
**R101A**	Forward: GTG CCA TCT CTA AAG GTG CTC AAG CCT GGC TGG ACC TCG GTA TTG TTC G<br>Reverse: CGA ACA ATA CCG AGG TCC AGC CAG GCT TGA GCA CCT TTA GAG ATG GCA C
**W102A**	Forward: CAT CTC TAA AGG TGC TCA ACG CGC GCT GGA CCT CGG TAT TGT TCG<br>Reverse: CGA ACA ATA CCG AGG TCC AGC GCG CGT TGA GCA CCT TTA GAG ATG
**W102F**	Forward: GTG CCA TCT CTA AAG GTG CTC AAC GCT TTC TGG ACC TCG GTA TTG TTC G<br>Reverse: CGA ACA ATA CCG AGG TCC AGA AAG CGT TGA GCA CCT TTA GAG ATG GCA C
**R109A**	Forward: CTG GCT GGA CCT CGG TAT TGT TGC TTT TCA GCC GTC GGA AAT TG<br>Reverse: CAA TTT CCG ACG GCT GAA AAG CAA CAA TAC CGA GGT CCA GCC AG
**Q111A**	Synthesized by Azenta Biosciences - Genewiz. Nucleotide sequence changed from CAG to GCG.
**E114A**	Forward: CTG GAC CTC GGT ATT GTT CGT TTT CAG CCG TCG GCA ATT GCC AAA ATA GCC GTA CCA CTG<br>Reverse: CAG TGG TAC GGC TAT TTT GGC AAT TGC CGA CGG CTG AAA ACG AAC AAT ACC GAG GTC CAG
**K117I**	Forward: TAT TGT TCG TTT TCA GCC GTC GGA AAT TGC CAT AAT AGC CGT ACC ACT GAT GGT TGC G<br>Reverse: CGC AAC CAT CAG TGG TAC GGC TAT TAT GGC AAT TTC CGA CGG CTG AAA ACG AAC AAT AC
**K117R**	Forward: CGT TTT CAG CCG TCG GAA ATT GCC CGA ATA GCC GTA CCA CTG ATG GTT GCG<br>Reverse: CGC AAC CAT CAG TGG TAC GGC TAT TCG GGC AAT TTC CGA CGG CTG AAA ACG
**K117N**	Forward: CAG CCG TCG GAA ATT GCC AAC ATA GCC GTA CCA CTG<br>Reverse: CAG TGG TAC GGC TAT GTT GGC AAT TTC CGA CGG CTG
**C133A**	Forward: CGC GCT TTA TCA ACC GCG ACG TTG CCC CGC CAT CGT TGA AGA ACA CTG<br>Reverse: CAG TGT TCT TCA ACG ATG GCG GGG CAA CGT CGC GGT TGA TAA AGC GCG
**D159V**	Forward: GCC CAC GCT GCT GGT GGC TGC ACA GCC TGT CCT GGG AAC ATC AAT CCT CGT TGC G<br>Reverse: CGC AAC GAG GAT TGA TGT TCC CAG GAC AGG CTG TGC AGC CAC CAG CAG CGT GGG C
**R210A**	Forward: CTG ATG CAT GAT TAC CAG CGC CAG GCC GTA ATG ATG CTC CTG GAC CCG G<br>Reverse: CCG GGT CCA GGA GCA TCA TTA CGG CCT GGC GCT GGT AAT CAT GCA TCA G
**E255A**	Forward: CTC AGT CAC AGC TTG AAT TTC TCC CCG CAC GCC ATA CTG ACT TTA TCT TCG CGG TAC TG<br>Reverse: CAG TAC CGC GAA GAT AAA GTC AGT ATG GCG TGC GGG GAG AAA TTC AAG CTG TGA CTG AG
**P257G**	Synthesized by Azenta Biosciences - Genewiz. Nucleotide sequence changed from CCC to GGC.
**H260A**	Forward: TCA CAG CTT GAA TTT CTC CCC GAA CGC GCT ACT GAC TTT ATC TTC GCG GTA CTG GC<br>Reverse: GCC AGT ACC GCG AAG ATA AAG TCA GTA GCG CGT TCG GGG AGA AAT TCA AGC TGT GA

**T261S**	Forward: GAA TTT CTC CCC GAA CGC CAT GCT GAC TTT ATC TTC GCG GTA CTG<br>Reverse: CAG TAC CGC GAA GAT AAA GTC AGC ATG GCG TTC GGG GAG AAA TTC
**D262A**	Forward: CCC GAA CGC CAT ACT GCC TTT ATC TTC GCG GTA<br>Reverse: TAC CGC GAA GAT AAA GGC AGT ATG GCG TTC GGG
**E270A**	Forward: GCC ATA CTG ACT TTA TCT TCG CGG TAC TGG CGG CAG AGC TGG GAT TAG TGG GCA TTC<br>Reverse: GAA TGC CCA CTA ATC CCA GCT CTG CCG CCA GTA CCG CGA AGA TAA AGT CAG TAT GGC
**S344A**	Synthesized by Azenta Biosciences - Genewiz. Nucleotide sequence changed from TCG to GCG.
**Q476C***	Forward: AAA GTC GAT AAC GTG TGC CAA ACG CTG GAC GCT<br>Reverse: AGC GTC CAG CGT TTG GCA CAC GTT ATC GAC TTT
**F401STOP - RodA Only**	Synthesized by Azenta Biosciences - Genewiz.
**G452STOP RodA and PBP2 helix only**	Synthesized by Azenta Biosciences - Genewiz.

Amino acid sequence of the RodA-PBP2 fusion construct:

MTDNPNKKTFWDKVHLDPTMLLILLALLVYSALVIWSASGQDIGMMERKIGQIAMGLV
IMVVMAQIPPRVYEGWAPYLYIICIILLVAVDAFGAISKGAQRWLDLGIVRFQPSEIAKIA
VPLMVARFINRDVCPPSLKNTGIALVLIFMPTLLVAAQPDLGTSILVALSGLFVLFLSGLS
WRLIGVAVVLVAAFIPILWFFLMHDYQRQRVMMLLDPESDPLGAGYHIIQSKIAIGSGGL
RGKGWLHGTQSQLEFLPERHTDFIFAVLAEELGLVGILILLALYILLIMRGLWIAARAQTT
FGRVMAGGLMLILFVYVFVNIGMVSGILPVVGVPLPLVSYGGSALIVLMAGFGIVMSIHT
HRKMLSKSVTSGSGSGSKLQNSFRDYTAESALFVRRALVAFLGILLLTGVLIANLYNLQI
VRFTDYQTRSNENRIKLVPIAPSRGIIYDRNGIPLALNRTIYQIEMMPEKVDNVQQTLDAL
RSVVDLTDDDIAAFRKERARSHRFTSIPVKTNLTEVQVARFAVNQYRFPGVEVKGYKRR
YYPYGSALTHVIGYVSKINDKDVERLNNDGKLANYAATHDIGKLGIERYYEDVLHGQT
GYEEVEVNNRGRVIRQLKEVPPQAGHDIYLTLDLKLQQYIETLLAGSRAAVVVTDPRTG
GVLALVSTPSYDPNLFVDGISSKDYSALLNDPNTPLVNRATQGVYPPASTVKPYVAVSA
LSAGVITRNTTLFDPGWWQLPGSEKRYRDWKKWGHGRLNVTRSLEESADTFFYQVAY
DMGIDRLSEWMGKFGYGHYTGIDLAEERSGNMPTREWKQKRFKKPWYQGDTIPVGIG
QGYWTATPIQMSKALMILINDGIVKVPHLLMSTAEDGKQVPWVQPHEPPVGDIHSGYW
ELAKDGMYGVANRPNGTAHKYFASAPYKIAAKSGTAQVFGLKANETYNAHKIAERLR
DHKLMTAFAPYNNPQVAVAMILENGGAGPAVGTLMRQILDHIMLGDNNTDLPAENPA
VAAAEDH

Supplementary References

1. Levy, N. *et al.* Structural Basis for E. coli Penicillin Binding Protein (PBP) 2 Inhibition, a Platform for Drug Design. *Journal of Medicinal Chemistry* **62**, 4742-4754 (2019).
2. Wu, Q. *et al.* Protein contact prediction using metagenome sequence data and residual neural networks. *Bioinformatics* **36**, 41-48 (2020).
3. Crooks, G.E., Hon, G., Chandonia, J.M. & Brenner, S.E. WebLogo: a sequence logo generator. *Genome Res* **14**, 1188-1190 (2004).
4. Steinegger, M. & Söding, J. MMseqs2 enables sensitive protein sequence searching for the analysis of massive data sets. *Nature Biotechnology* **35**, 1026-1028 (2017).
5. Worswick, S.G., Spencer, J.A., Jeschke, G. & Kuprov, I. Deep neural network processing of DEER data. *Sci Adv* **4**, eaat5218 (2018).
6. Jeschke, G. *et al.* DeerAnalysis2006—a comprehensive software package for analyzing pulsed ELDOR data. *Applied Magnetic Resonance* **30**, 473-498 (2006).

Source data:

Supplementary Fig. 1c

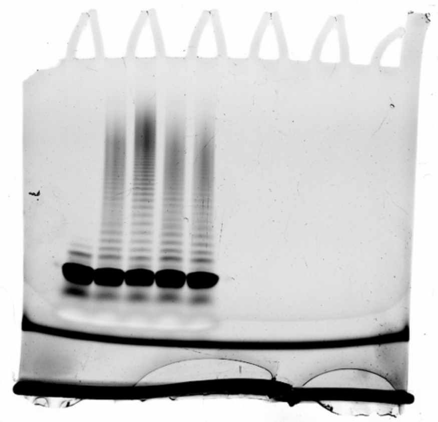

Supplementary Fig. 1d

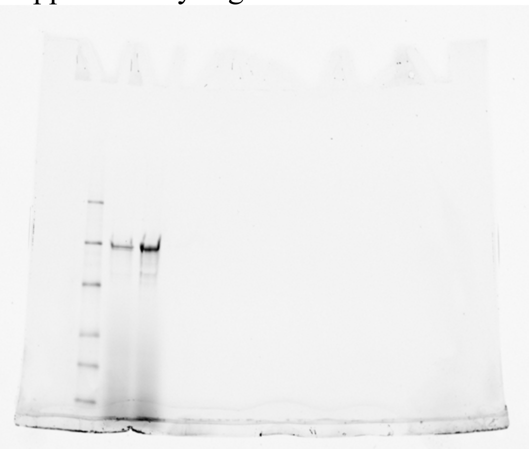

Supplementary Fig. 1e

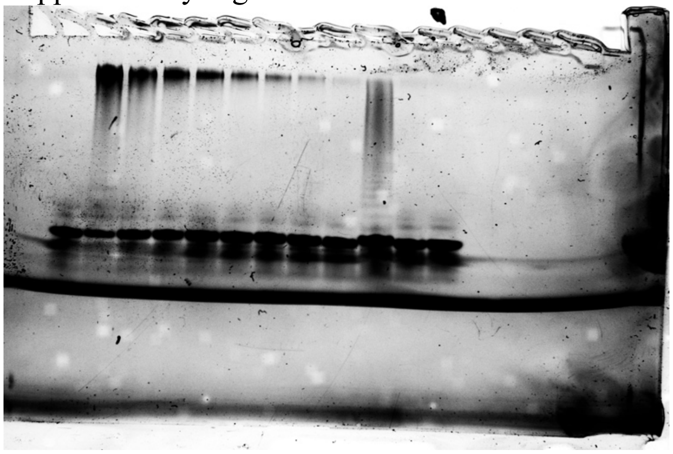

Supplementary Fig. 1f

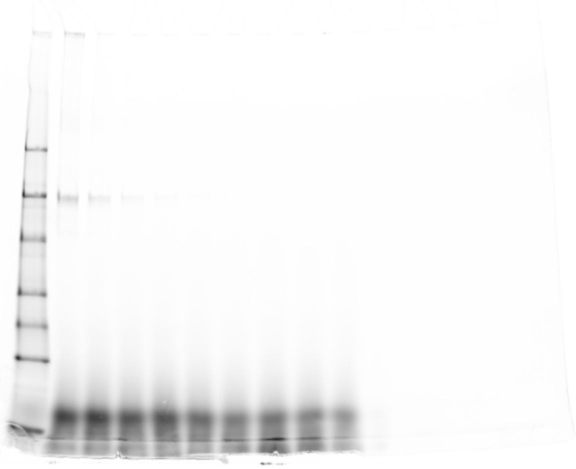

Supplementary Fig. 1g

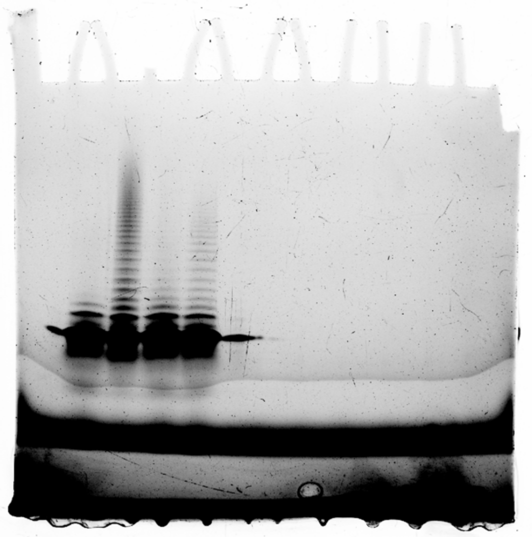

Supplementary Fig. 1h

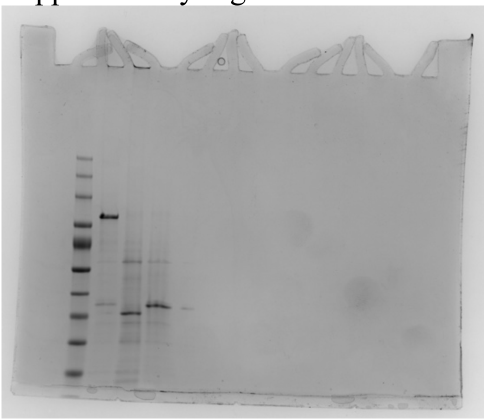

Supplementary Fig. 1i

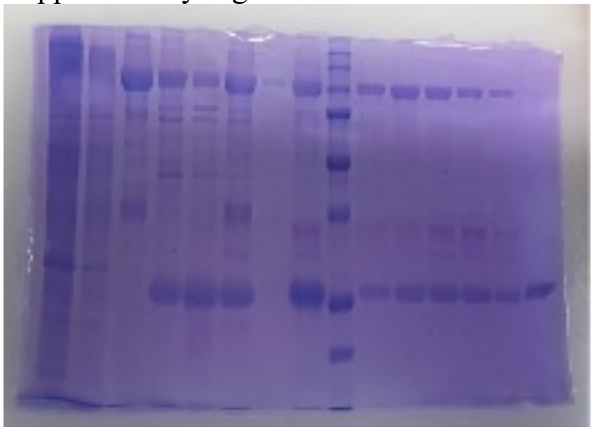

Supplementary Fig. 7a

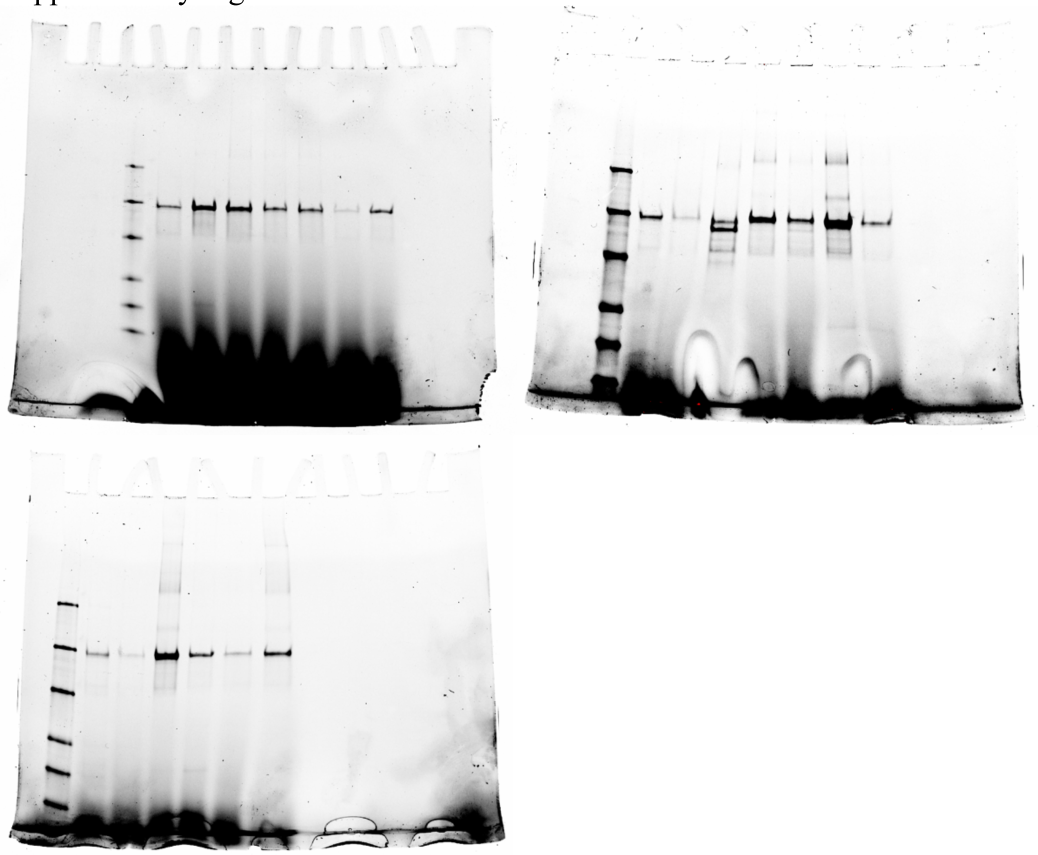

Supplementary Fig. 7b

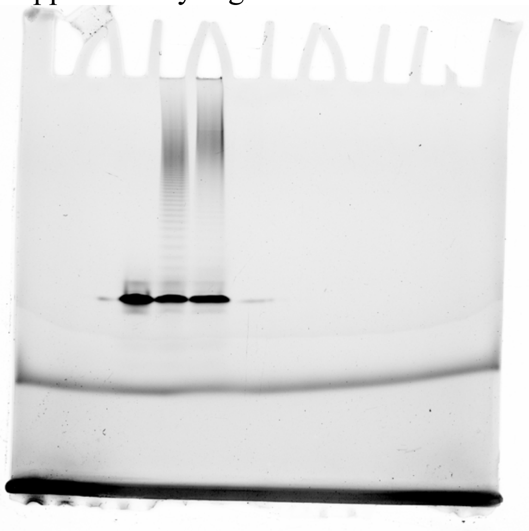

Supplementary Fig. 9a

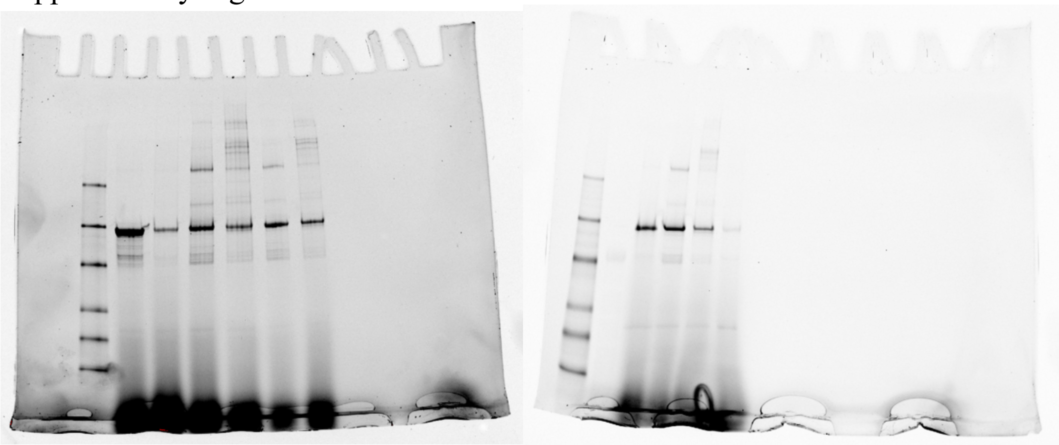

Supplementary Fig. 9b

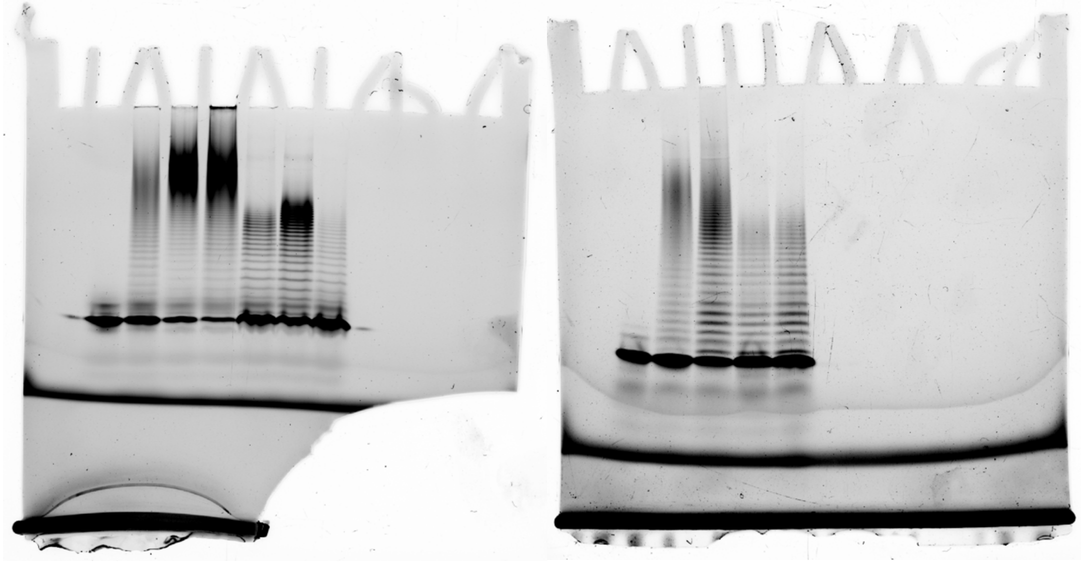

Supplement: Supplementary file 1 — Supplementary Information [file 41467_2023_40483_MOESM1_ESM.pdf]
