# Supplementary material for: Structural basis of peptidoglycan synthesis by E. coli RodA-PBP2 complex
Source: Nat Commun. 2023 Aug 24;14:5151. doi: 10.1038/s41467-023-40483-8 (PMC10449877; doi:10.1038/s41467-023-40483-8)
Supplement: Supplementary file 3 — Description of Additional Supplementary Files [file 41467_2023_40483_MOESM3_ESM.pdf]

File name: Supplementary Movie 1

Description: Molecular animation of the growing glycan strand. The movie illustrates the multiple steps that occur during elongation of the glycan strand. In each instance a Lipid II molecule binds to cavity B to initiate the process, with either Lipid II or a longer peptidoglycan polymer found in cavity A. After each reaction, the product, which forms in cavity B moves to cavity A. We hypothesize that this is via a channel within RodA. This pushes the growing peptidoglycan towards the transpeptidation domain within PBP2. The Und-PP product (not shown) is released from cavity A either between TM7 and the core of RodA or under the JM helices into the membrane. RodA-PBP2 is shown as surface representation in grey. The Und-PP lipid is represented in black sticks and the disaccharides with the pentapeptide stem attached as sticks in different colors.
