# Supplementary material for: Structural basis of peptidoglycan synthesis by E. coli RodA-PBP2 complex
Source: Nat Commun. 2023 Aug 24;14:5151. doi: 10.1038/s41467-023-40483-8 (PMC10449877; doi:10.1038/s41467-023-40483-8)
Supplement: Supplementary file 5 — Reporting Summary [file 41467_2023_40483_MOESM5_ESM.pdf]

nature portfolio

Corresponding author(s): Linda Columbus, Jonathan Dworkin, Phillip Stansfeld, David Roper and Filippo Mancia

Last updated by author(s): Jul 19, 2023

Reporting Summary

Nature Portfolio wishes to improve the reproducibility of the work that we publish. This form provides structure for consistency and transparency in reporting. For further information on Nature Portfolio policies, see our Editorial Policies and the Editorial Policy Checklist.

Statistics

For all statistical analyses, confirm that the following items are present in the figure legend, table legend, main text, or Methods section.

n/a	Confirmed	
☐	☒	The exact sample size (*n*) for each experimental group/condition, given as a discrete number and unit of measurement
☒	☐	A statement on whether measurements were taken from distinct samples or whether the same sample was measured repeatedly
☒	☐	The statistical test(s) used AND whether they are one- or two-sided<br>*Only common tests should be described solely by name; describe more complex techniques in the Methods section.*
☒	☐	A description of all covariates tested
☒	☐	A description of any assumptions or corrections, such as tests of normality and adjustment for multiple comparisons
☐	☒	A full description of the statistical parameters including central tendency (e.g. means) or other basic estimates (e.g. regression coefficient) AND variation (e.g. standard deviation) or associated estimates of uncertainty (e.g. confidence intervals)
☒	☐	For null hypothesis testing, the test statistic (e.g. *F*, *t*, *r*) with confidence intervals, effect sizes, degrees of freedom and *P* value noted<br>*Give P values as exact values whenever suitable.*
☒	☐	For Bayesian analysis, information on the choice of priors and Markov chain Monte Carlo settings
☒	☐	For hierarchical and complex designs, identification of the appropriate level for tests and full reporting of outcomes
☒	☐	Estimates of effect sizes (e.g. Cohen's *d*, Pearson's *r*), indicating how they were calculated

Our web collection on statistics for biologists contains articles on many of the points above.

Software and code

Policy information about availability of computer code

Data collection	Leginon
Data analysis	For Cryo-EM data processing the following software were used: CryoSparc v2.12 and v3.2<br>For model building the following software were used: Namdinator, Coot v.0.9.8.7, Phenix v1.19.2, Xtal Pred server, Voss Volume Voxelator, UCSF Chimera v1.15, ChimeraX v1.2, pymol 2.4<br>For sequence alignment the following software was used: MMseqs2, Weblogo3, GREMLIN, BLAST, consurf<br>For molecular dynamics simulations the following software was used: modeller 9.24, vmd 1.9.4, GROMACS 2021, memembed, Martini 2, Martini 3, martinize2, CHARMM36m, CG2AT, ORCA program v5.0.3, PyLipID<br>For DEER data analysis the following software were used: DEERAnalysis 2019, DEERAnalysis 2022, MMM 2021.4, and PyMol v2.5.2<br>Gel imagine software: Biorad imaging capture software.

For manuscripts utilizing custom algorithms or software that are central to the research but not yet described in published literature, software must be made available to editors and reviewers. We strongly encourage code deposition in a community repository (e.g. GitHub). See the Nature Portfolio guidelines for submitting code & software for further information.

Data

Policy information about availability of data

All manuscripts must include a data availability statement. This statement should provide the following information, where applicable:
- Accession codes, unique identifiers, or web links for publicly available datasets
- A description of any restrictions on data availability
- For clinical datasets or third party data, please ensure that the statement adheres to our policy

The density maps have been deposited into the Electron Microscopy Data Bank (EMDB), with accession code EMD-41299 (https://www.ebi.ac.uk/pdbe/entry/emdb/EMD-41299). The model has been deposited in the Protein Data Bank (PDB), with accession code 8TJ3 (http://doi.org/ 10.2210/pdb8tj3/pdb).

Human research participants

Policy information about studies involving human research participants and Sex and Gender in Research.

Reporting on sex and gender	N/A
Population characteristics	N/A
Recruitment	N/A
Ethics oversight	N/A

Note that full information on the approval of the study protocol must also be provided in the manuscript.

Field-specific reporting

Please select the one below that is the best fit for your research. If you are not sure, read the appropriate sections before making your selection.

☒ Life sciences ☐ Behavioural & social sciences ☐ Ecological, evolutionary & environmental sciences

For a reference copy of the document with all sections, see nature.com/documents/nr-reporting-summary-flat.pdf

Life sciences study design

All studies must disclose on these points even when the disclosure is negative.

Sample size	Number of micrographs collected have been disclosed
Data exclusions	No data is excluded from the study
Replication	All electrophoresis experiments of Lipid II polymerization was repeated at least three times to assure reproducibility
Randomization	Randomization was not applicable for this study
Blinding	Blinding was not applicable for this study

Reporting for specific materials, systems and methods

We require information from authors about some types of materials, experimental systems and methods used in many studies. Here, indicate whether each material, system or method listed is relevant to your study. If you are not sure if a list item applies to your research, read the appropriate section before selecting a response.

Materials & experimental systems

n/a	Involved in the study
☒	☐ Antibodies
☒	☐ Eukaryotic cell lines
☒	☐ Palaeontology and archaeology
☒	☐ Animals and other organisms
☒	☐ Clinical data
☒	☐ Dual use research of concern

Methods

n/a	Involved in the study
☒	☐ ChIP-seq
☒	☐ Flow cytometry
☒	☐ MRI-based neuroimaging
