# Supplementary figures and images for: Structural basis of peptidoglycan synthesis by E. coli RodA-PBP2 complex
Source: Nat Commun. 2023 Aug 24;14:5151. doi: 10.1038/s41467-023-40483-8 (PMC10449877; doi:10.1038/s41467-023-40483-8)

**Figure 2e**


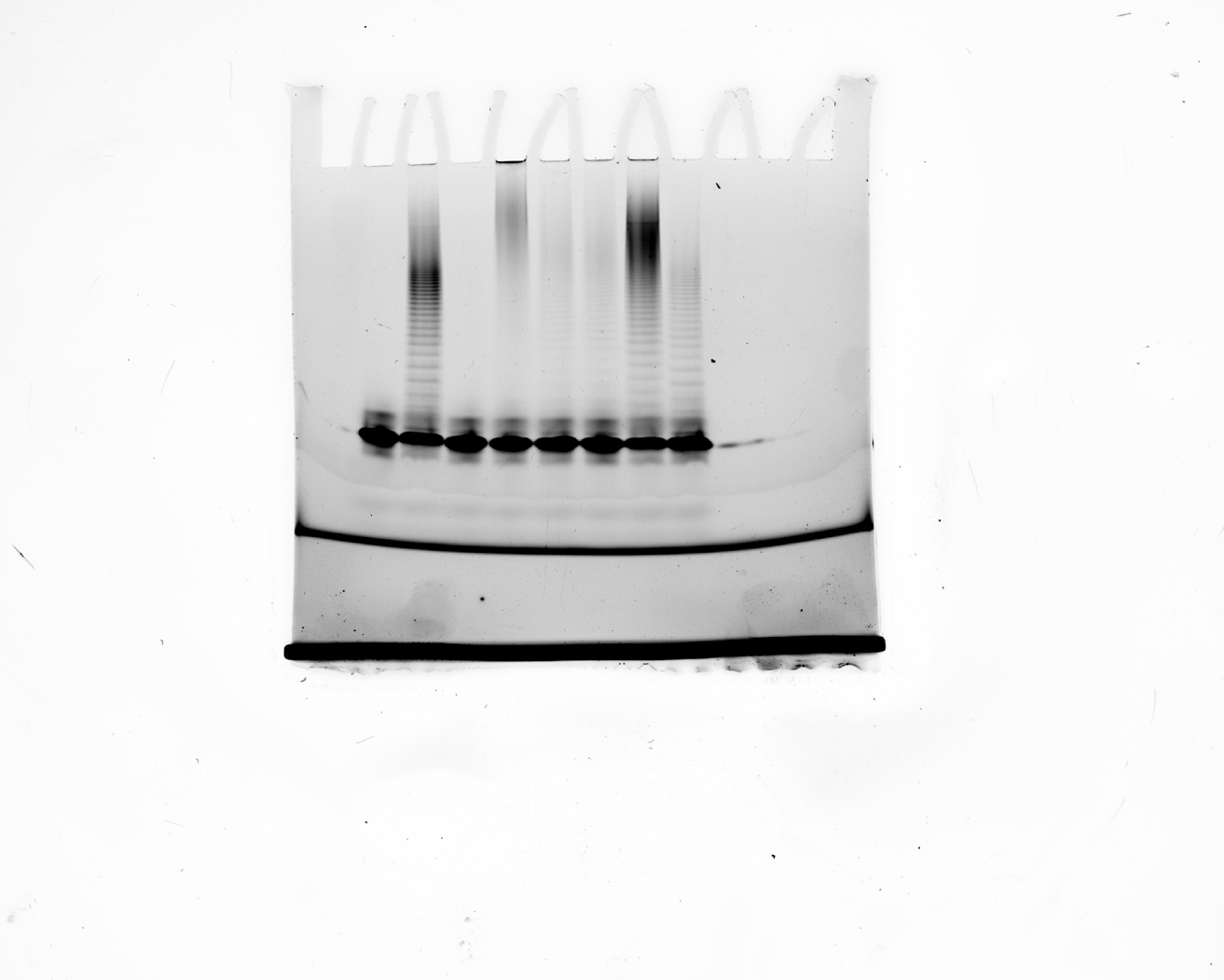

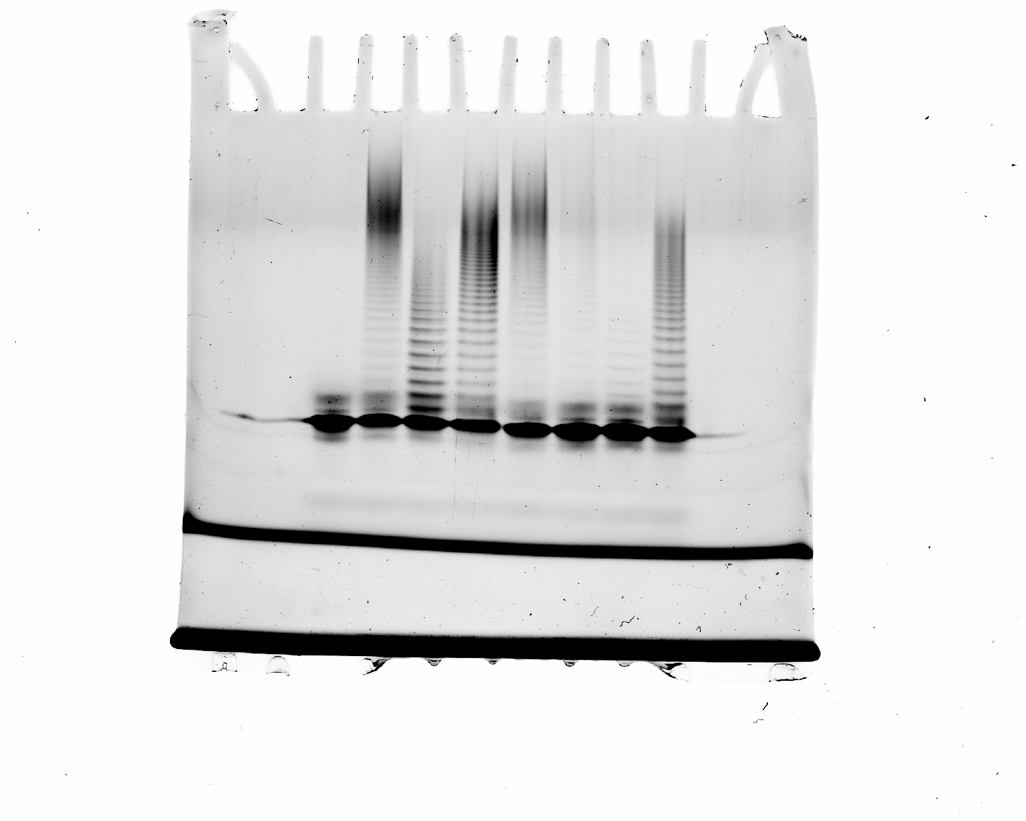


**Figure 4b**


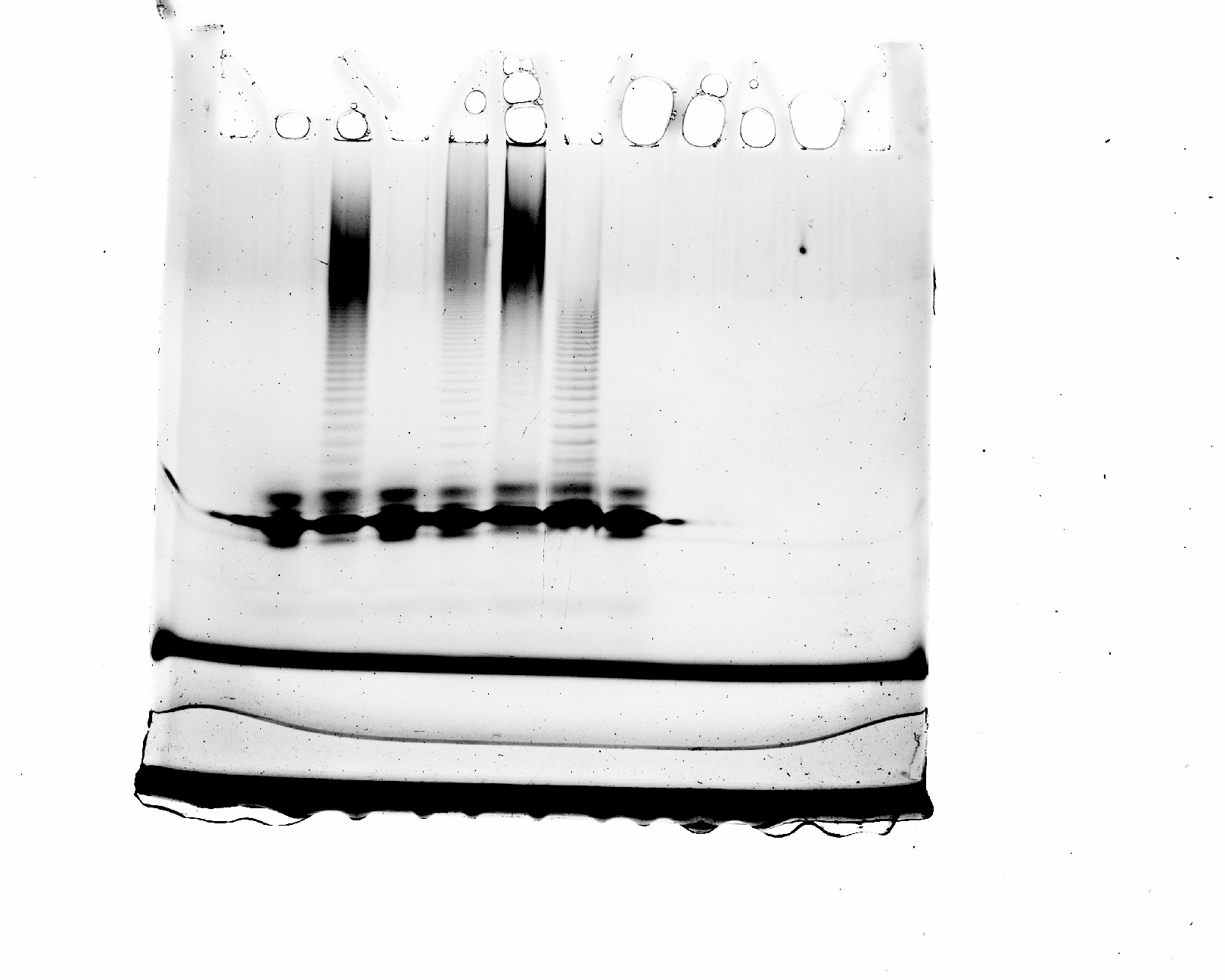

Supplement: Supplementary file 6 — Source Data [file 41467_2023_40483_MOESM6_ESM.docx]
